# Addressing Discrepancies between Experimental and Computational Procedures

**DOI:** 10.3390/biology10060536

**Published:** 2021-06-15

**Authors:** Milan Toma, Satvinder K. Guru, Wayne Wu, May Ali, Chi Wei Ong

**Affiliations:** 1 Serota Academic Center (Room 138), New York Institute of Technology, Department of Osteopathic Manipulative Medicine, College of Osteopathic Medicine, Northern Boulevard, P.O. Box 8000, Old Westbury, NY 11568, USA; sguru@nyit.edu (S.K.G.); ywu71@nyit.edu (W.W.); mali60@nyit.edu (M.A.); 2 Agency for Science, Technology and Research (A*STAR), Institute of High Performance Computing (IHPC), 1 Fusionopolis Way, #16-16 Connexis, Singapore 138632, Singapore; Ong_Chi_Wei@ihpc.a-star.edu.sg

**Keywords:** fluid–structure interaction, heart valve, comprehensive computational model, smooth particle hydrodynamics, chordal structure, chordae tendineae, fixation, inverse finite element

## Abstract

**Simple Summary:**

This technical note addresses the need to consider uncertainties when using experimental procedures to extract a geometry that is consequently used for computational simulations. Many uncertainties enter the process in both the experimental and computational techniques.

**Abstract:**

Imaging subject-specific heart valve, a crucial step to its design, has experimental variables that if unaccounted for, may lead to erroneous computational analysis and geometric errors of the resulting model. Preparation methods are developed to mitigate some sources of the geometric error. However, the resulting 3D geometry often does not retain the original dimensions before excision. Inverse fluid–structure interaction analysis is used to analyze the resulting geometry and to assess the valve’s closure. Based on the resulting closure, it is determined if the geometry used can yield realistic results. If full closure is not reached, the geometry is adjusted adequately until closure is observed.

## 1. Introduction

Leonardo da Vinci’s later work (≈1506 until his death in 1519) encompasses studies on the heart (among other organs) from the perspective of an architect–engineer and is permeated with recognition as to how mechanics relate to human pathology. Half a millennium later, with the progress of scientific research and development in the field of mechanics, we enjoy new ways to study how mechanics relate to human pathology. Moreover, the urgency to continue exploring the mechanics of heart valves and new products and procedures related to their repair/replacement continues to be relevant. Artificial heart valves and their delivery systems are so complex that they often culminate in subsequent recalls of the related products [1,2]. The use of computational analyses is increasingly adopted to assist in developing these products to decrease the likelihood of recalling them in the future.

The computational assessments of these systems are associated with their own set of challenges [3]. For example, the lack of realistic human models limits our ability to simulate realistic boundary conditions. Ideally, the 3D models would be acquired from an active human body. Unfortunately, the imaging modalities currently available to be used in vivo do not always possess the ability to capture every geometrical detail to guarantee that models acquired that way would realistically represent the actual organ. It can be even argued that such technology will never be developed. Hence, in vitro procedures have been developed to more accurately capture every detail. However, these procedures, too, are associated with their own set of challenges.

This study uses a heart valve (HV) scanned with micro-computed tomography (*μ*CT) and assumes that a geometric error occurs in the resulting 3D model acquired from the medical images. For example, as described in [4], the surface tension caused by residual moisture on the valve apparatus appears to be one of them. This phenomenon causes a “bunching” effect on the leaflets and chordae tendineae, which yields 3D datasets in which the leaflets appear smaller and thicker, and the delicate chordal trees appear as bulky chordae with minimal branching. More severe folding can be observed in the thin, aqueous marginal section than in the thicker, more collagenous basal section of the anterior leaflet.

It is hypothesized that the well-characterized stiffening effects of glutaraldehyde fixation [5] would counteract this bunching effect caused by surface tension. Therefore, in an attempt to capture the physiological diastolic detail of the heart valves and prevent distortions, fixation techniques and preparation methods were developed [4,6]. These preparation methods mitigate some sources of geometric error when scanning HV with *μ*CT in air. In addition, many more experimental and computational uncertainties have to be considered. As shown in [7], even just the user’s choice regarding image processing techniques used to process the same set of medical images yields different resulting geometries. In this case, the uncertainties can be counterbalanced computationally, as demonstrated in this paper. Due to the presence of many valvular heart diseases, the HV model with realistic dimensions, and little to no interference by the uncertainties, is essential. In clinical settings, accurate and realistic HV geometries can potentially help surgeons better evaluate the outcomes of specific valvular procedures.

## 2. Materials and Methods

The computational platform for establishing HV geometry is based on *μ*CT datasets, followed by image processing, mesh generation and, finally, valve closure simulation using a fluid–structure interaction (FSI) approach [8]. The following sections briefly describe the process used to develop the 3D HV model and the numerical analysis used to simulate the closure of the HV.

### 2.1. Developing a 3D Heart Valve Model

**Figure 1:**
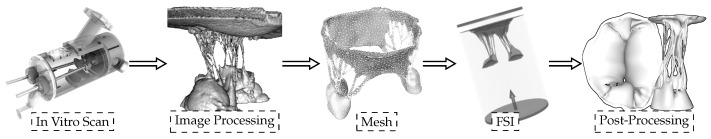
Sequential experimental techniques to develop the 3D model with preserved valve-specific geometry.

To develop a “valve-specific” geometry for the FSI simulation of the heart valve, a locally sourced ovine mitral valve is obtained to avoid rigor mortis. The process of model development includes: (1) CT scanning, (2) image processing to develop a 3D model, and (3) high quality, robust mesh generation (Figure 1). As explained in [4], a pulsatile cylindrical left heart simulator (CLHS) is sealed and inserted into a closed loop with a steady-flow pump (Figure 1 left) [9], which maintains a flow rate of approximately 20 L/m through the CLHS and across the MV. That opens the MV leaflets and spreads out the chordae tendineae, while the glutaraldehyde solution fixed the tissue. The CLHS is then dismounted from this steady-flow loop, drained and rinsed, and imaged. Consequently, an FSI analysis to evaluate the opening and closing of the valve is performed. A detailed description of how the model was developed can be found in [8].

### 2.2. Fluid–Structure Interaction Simulations

**Figure 2:**
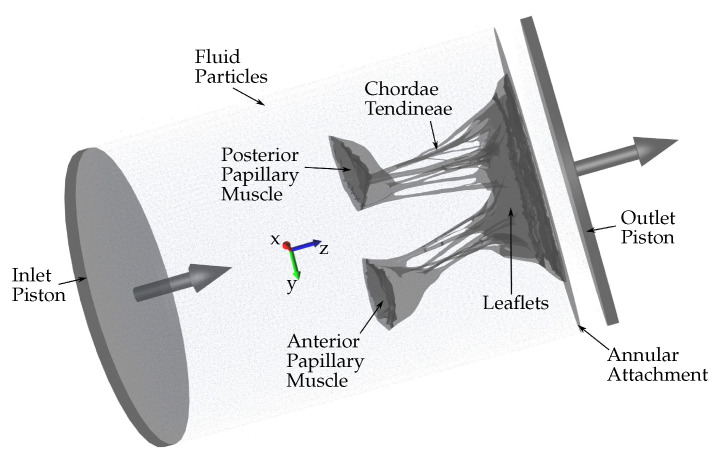
Schematic showing the fluid particles confined in a pipe-like rigid structure surrounding the HV model. Prescribed velocity boundary conditions are applied to the open ends via the use of moving pistons in z-direction.

Several approaches can be adopted to simulate the HV function using FSI. In this study, an approach based on smoothed particle hydrodynamics (SPH) is adopted, i.e., the fluid domain is modeled as a collection of discrete particles [10]. The fluid particles confined in a pipe-like rigid structure surrounding the HV model can be seen in Figure 2. A detailed explanation of this approach used to simulate mitral valve function can be found in [11].

The combination of SPH methods (used to simulate the fluid domain flow) with a high-order finite element method (used to simulate the solid domain deformations) is ideal for simulating FSI when complex geometries are included [12]. Using SPH methods provides numerical stability because the contact between the solid and fluid domains is easily defined numerically. Moreover, SPH is highly parallelizable. Hence, it is possible to run FSI simulations that are numerically stable, precise, and parallelized on a standard GPU workstation (as opposed to large supercomputers), which do not require the use of simplified geometries and possess a runtime of hours or days rather than weeks and months. Fluid motion and boundary interaction are solved with the IMPETUS Afea SPH Solver^®^ while large deformation in the solid domain, i.e., the HV, is simultaneously solved with the IMPETUS Afea Solver^®^. Both the solvers use GPU parallel processing. The contact, i.e., particle to structure contact, is very simple, which is why SPH is ideal for these complex applications. It can easily account for movement in any direction, unlike finite element fluid solvers, which involve more complicated contact and usually require re-meshing of the fluid domain during the simulation. The ability of IMPETUS to achieve a very high resolution in terms of particle density results in a very accurate particle to structure contact, especially in regards to a structure as detailed and complex as MV.

### 2.3. Counterbalancing the Uncertainties

As mentioned above, one of the many uncertainties to consider, the “bunching” effect of leaflets and chordae tendineae, happens when the excised HV specimen is mounted onto the annulus plate of pulsatile CLHS [9]. Papillary muscle positions are adjusted to achieve healthy closure according to commonly used parameters (anterior leaflet occupying 23 of the anterior–posterior diameter, coaptation height of 3–5 mm, minimal tenting and prolapse, mitral regurgitation volume < 5 mL). However, after the chamber is dismounted from the pulse duplicator in preparation for *μ*CT imaging, the “bunching” effect can be observed.

The “bunching” effect can be partially counteracted by using appropriate fixation techniques and preparation methods. Even then, after the medical images from the *μ*CT dataset are used to develop the “valve-specific” geometry, it has to be confirmed how effective these in vitro preparation methods are. However, the “bunching” effect is just one of many uncertainties to consider, many of which are unknown and, therefore, impossible to address using preparation methods.

Confirming the effectiveness of preparation methods used can be done in silico by performing an FSI simulation of the HV closure. Suppose the HV closure cannot be reached computationally. In that case, it is assumed that the preparation methods did not effectively counteract the uncertainties and the 3D model needs to be adjusted to further account for the discrepancies between the experimental and computational procedures. In this study, the model is elongated in the z-direction, and multiple FSI simulations are performed until the HV closure is observed and validated.

## 3. Results

**Figure 3:**
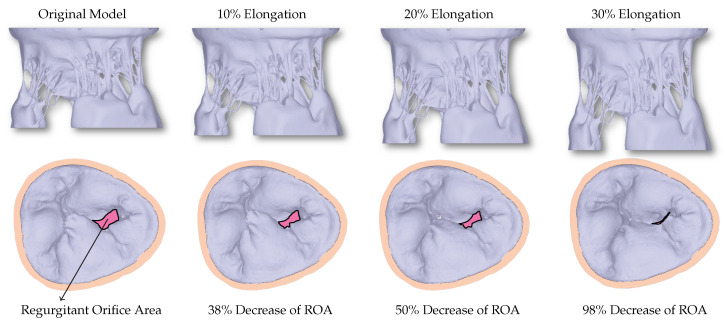
The first row shows the valves with different levels of elongation in order to correct for the uncertainties that occur during imaging of the HV. The second row shows the corresponding closure reached using the FSI simulations. A decrease in the regurgitant orifice area (ROA) can be observed with increased elongation.

The excised HV in experimental settings is adjusted appropriately to achieve healthy closure. However, before and during *μ*CT imaging, many uncertainties can occur during the exposure to air. Consequently, the original model developed directly from the *μ*CT dataset cannot achieve closure, and a large non-zero regurgitant orifice area (ROA) can be seen (Figure 3).

**Figure 4:**
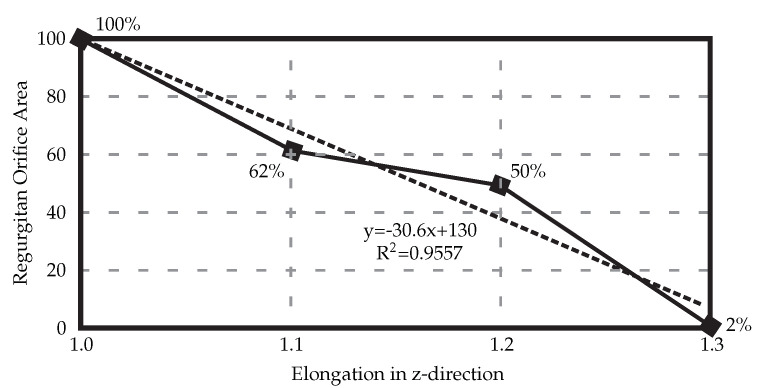
Elongating the resulting 3D HV model developed from the medical *μ*CT images results in decreased regurgitant orifice area. In this case, elongation of 30% is necessary to achieve full healthy closure.

When the HV model is elongated in the z-direction to counteract the uncertainties, a linear relationship between the elongation and ROA can be seen (Figure 4), with 30% elongation yielding healthy closure as observed in experimental settings before the exposure to the air.

**Figure 5:**
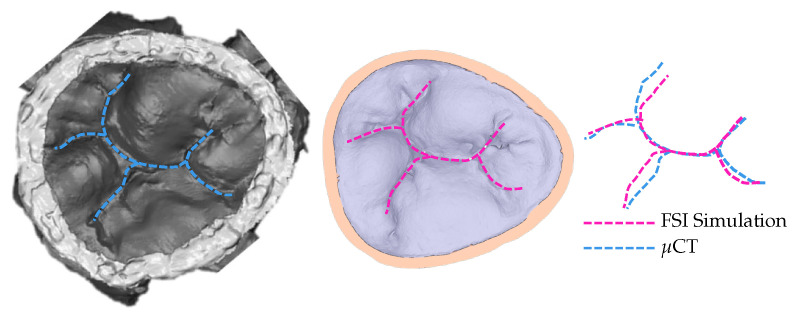
Coaptation lines from *μ*CT of closed HV and computational simulation are used to validate the resulting simulated closure. To achieve this closure, the model with 30% elongation, shown in Figure 3, is used.

To confirm that the achieved simulated closure, using the model with 30% elongation compared to the original model, matches the closure observed in experimental settings, the coaptation lines are compared (Figure 5). The same material property values are used for the entire model. Multiple, more precise material properties prescribed to appropriate parts of the model would yield coaptation lines with even better match [13].

## 4. Discussion

The use of computational analyses to assist the development of medical devices and procedures lacks realistic human models, limiting the ability to simulate realistic boundary conditions. Many new medical devices are not tested in a human environment before approval (it is highly costly when utilizing human testing). Even when approved, the number of recalls is increasing. Hence, developing well-validated computational (in silico) testing that assists in the development of novel medical devices is desirable. More effective medical devices yield improved patient quality of life and ultimately decreased cost of treatment.

When HV is excised from the heart for further processing, it no longer possesses its properties compared to when it is in a living, beating heart. (That is also the case for any other organ after excision from a living body.) The tissue of HV is susceptible and quickly suffers changes when not submerged in a liquid. For example, it is known that HV tissue experiences the “bunching” effect when exposed to air, causing leaflets to appear smaller and thicker while chordae tendineae appear bulkier with minimal branching. In addition to the changes in the tissue when exposed to air, many more uncertainties enter the process from excision to securing a realistic 3D model used for predictive simulations. Even when the same dataset is used, different users will produce differing geometries [7]. When exact quantification of how much the HV changes is not known, the inverse finite element method (or, in this case, the FSI method) is used to assess the level of uncertainties. By achieving healthy full closure and validating it against in vitro observation (i.e., *μ*CT), it is confirmed that, for this specific specimen and methods used, the 30% elongation of the original model (i.e., developed from the *μ*CT dataset) represents more realistic HV dimensions. This computational platform has been previously validated and used in several of our studies. Our group has studied the mitral valve function in both healthy [4,8,11] and diseased states [14,15,16]. Other HVs, namely the tricuspid valve, were also studied using the same methods [13].

## 5. Conclusions

In most cases, especially when the geometry being extracted (to be processed and used for computational simulations) is complex, in vitro and in silico procedures are accompanied with uncertainties. The entire process includes many experimental and numerical procedures, and every step introduces uncertainty. These uncertainties need to be carefully balanced to ensure that the resulting geometry and, consequently, the results of the computational simulations are realistic. In this study, with the procedures as described, elongation was used as the factor which, when adjusted, leads to more realistic geometry with HV closure validated against experimental data. However, depending on the procedures used in vitro and in silico, other factors might have to be considered. For example, the fixation techniques to prevent the “bunching” effect need to be studied to confirm their efficiency. On the in silico side, our choice of numerical algorithms used affects the resulting geometry. There is more work to be done exploring these uncertainties.

## Data Availability

The data presented in this study are available on request from the corresponding author.

## Abbreviations

The following abbreviations are used in this manuscript:

3D	3 dimensional
HV	Heart valve
*μ*CT	Micro-computed tomography
FSI	Fluid–structure interaction
SPH	Smoothed-particle hydrodynamics
GPU	Graphics processing unit
ROA	Regurgitant orifice area
